# Risk Factors for Lymph Node Metastasis in a Western Series of Patients with Distal Early Gastric Cancer

**DOI:** 10.3390/jcm13092659

**Published:** 2024-05-01

**Authors:** Maria Michela Chiarello, Serafino Vanella, Pietro Fransvea, Valentina Bianchi, Valeria Fico, Anna Crocco, Giuseppe Tropeano, Giuseppe Brisinda

**Affiliations:** 1 Unità Operativa di Chirurgia Generale, Dipartimento di Chirurgia, Azienda Sanitaria Provinciale, 87100 Cosenza, Italy; michela.chiarello@aspcs.it; 2 Unità Operativa di Chirurgia Generale e Oncologica, Azienda Ospedaliera di Rilevanza Nazionale San Giuseppe Moscati, 83100 Avellino, Italy; serafino.vanella@aornmoscati.it; 3 Unità Operativa di Chirurgia d’Urgenza e del Trauma, Dipartimento Scienze Mediche e Chirurgiche Addominali ed Endocrino Metaboliche, Istituto di Ricerca e Cura a Carattere Scientifico (IRCCS), Fondazione Policlinico Universitario Agostino Gemelli, 00168 Roma, Italy; pietro.fransvea@policlinicogemelli.it (P.F.); valentina.bianchi@guest.policlinicogemelli.it (V.B.); valeria.fico@policlinicogemelli.it (V.F.); giuseppe.tropeano@guest.policlinicogemelli.it (G.T.); 4 Unità Operativa di Chirurgia Oncologica Della Tiroide e Della Paratiroide, Istituto Nazionale Tumori, IRCCS Fondazione Pascale, 80100 Napoli, Italy; anna.crocco@istitutotumori.na.it; 5 Dipartimento Universitario di Medicina e Chirurgia Traslazionale, Università Cattolica del Sacro Cuore, 00168 Roma, Italy

**Keywords:** early gastric cancer, lymph node metastasis, lymphadenectomy, risk factors

## Abstract

**Background:** Assessment of potential lymph node metastasis is mandatory in the appropriate treatment of early gastric cancers. This study analysed factors associated with lymph node metastasis to identify differences between node-negative and node-positive patients and between T1a and T1b cancers. **Methods:** The clinicopathological features of 129 early gastric cancer patients who had undergone radical gastrectomy were analysed to identify predictive factors for lymph node metastasis. **Results:** Lymph node metastasis was detected in 76 (59.0%) patients. Node-positive patients were younger (58.1 ± 11.3 years) than those without metastasis (61.9 ± 9.6 years, *p* = 0.02). Greater tumour sizes were observed in patients with lymph node metastasis (3.6 ± 1.0 cm) compared to node-negative patients (1.9 ± 0.5 cm, *p* = 0.00001). Depressed form, ulceration, diffuse histological type, and undifferentiated lesions were more frequent in node-positive patients than in the node-negative group. Tumour size > 3.0 cm showed a correlation with lymph node metastasis in both T1a (*p* = 0.0001) and T1b (*p* = 0.006) cancer. The male sex (*p* = 0.006) had a significant correlation with lymph node metastasis in T1a cancer. Depressed appearance (*p* = 0.02), ulceration (*p* = 0.03), differentiation (*p* = 0.0001), diffuse type (*p* = 0.0002), and lower third location (*p* = 0.005) were associated with lymph node metastasis in T1b cancer. **Conclusions:** Tumour size > 3 cm, undifferentiated lesions, ulceration, diffuse type, lower third location, and submucosal invasion are risk factors for lymph node metastasis in early gastric cancer.

## 1. Introduction

Gastric cancer is a malignancy with great heterogenicity [1]. The application of the same standard to all patients in different conditions may lead to treatment bias [1,2,3,4]. In early gastric cancer (EGC), the depth of invasion is limited to the mucosa (T1a) and submucosa (T1b), regardless of lymph node (LN) involvement [3,5,6]. The lymphatic system plays an important role in the spread of gastric cancer and intramucosal growth is sufficient to cause lymph node metastasis (LNM) [7,8,9]. Although gastrectomy has been associated with superior survival compared with alternative procedures, endoscopic resection and limited surgery have been widely accepted as a curative therapy for small T1 cancers [10,11] in order to reduce perioperative complications and improve quality of life. In this scenario, a preoperative assessment of risk factors for LNM based on clinicopathological factors may be useful for optimal treatment planning [3,9,12]. It is a significant prognostic factor for EGC, because the disease is believed to be curable [2,13,14]. Although there has been substantial research on the prediction of risk factors for LNMs in EGC, no definitive criteria are available [15]. Risk factors for LNM are well established in Eastern countries [5,16,17]. Nevertheless, an analysis of predictive factors for LNMs in EGC remains uncommon in the West, where series of patients are smaller in comparison to the East [3,17,18,19,20]. Moreover, the risk of LNMs in Western patients with EGC is higher, probably due to a higher incidence of T1b cancers [21,22]. In these patients, radical surgery with formal LN dissection has become the standard treatment [3,23,24,25]. Therefore, it was noted that about 70–80% of patients undergo unnecessary extended LN dissection. The present study was designed to evaluate the factors that can predict the presence of LNMs. The primary aim of this study is to evaluate the factors determining the prevalence of LNMs. An evaluation of factors is performed to identify the difference between T1a and T1b cancers and between node-negative and LNM patients.

## 2. Materials and Methods

A retrospective analysis was conducted on EGC patients who had undergone D2 radical gastrectomy at Fondazione Policlinico Universitario “A Gemelli” IRCCS and at the Department of Surgery, Azienda Sanitaria Provinciale Crotone, over a 15-year period. This study followed the STROBE reporting guidelines [26]. This study was conducted in accordance with the Declaration of Helsinki. All patients provided written consent before the surgical procedures.

Patients were eligible if they met the following inclusion criteria: 1. middle third or lower third EGC; 2. lymphadenectomy with more than 16 LNs harvested. Patients with advanced gastric cancer or neoplasms other than gastric adenocarcinoma, EGC occurring in the esophagogastric junction, in the upper third, and EGC of the gastric stump, those undergoing endoscopic resection or neoadjuvant treatment, or those with missing histopathological data were excluded.

EGC was divided into T1a and T1b according to the depth of invasion, as indicated in the AJCC Cancer Staging system [27]. The location of the tumour was defined according to the JGCA classification. The maximum diameter of the tumour was recorded as tumour size. Macroscopic type included elevated and depressed. Histopathological classification followed the Lauren criteria [28]. The intestinal and mixed type were grouped as intestinal tumours. Tumour histology was classified into a differentiated type, which included papillary adenocarcinoma and well- or moderately differentiated adenocarcinoma, and an undifferentiated type, which included poorly or undifferentiated adenocarcinoma, signet ring cell carcinoma, and mucinous carcinoma. Lesions with ulceration or scarring from previous ulceration were regarded as ulcerated lesions.

Total or subtotal gastrectomy always included omentectomy and cholecystectomy. Cholecystectomy was performed both in patients with documented gallstones and in patients without concomitant biliary disease. This was because in all patients, for reconstruction, the Roux-en-Y technique was performed. The JGCA guidelines were used for the definition of D2 lymphadenectomy [29]. N stage was classified into four levels based on the number of LNMs, as follows: N0, no regional LMN; N1, 1 to 2 LNMs; N2, 3 to 6 LNMs; N3, >7 LNMs. Each LN station was removed, classified, and then submitted to histopathological examination, as specified elsewhere [6,30].

The evaluated parameters included patient age, sex, tumour size, tumour site at endoscopy, Lauren’s histological type, tumour differentiation, presence of ulceration, macroscopic appearance, depth, N stage, and number of retrieved LNs and LNMs. Subgroups were identified in relation to age (≤65 years and >65years), tumour size (≤3.0 cm and >3.0 cm), longitudinal localisation (GRE—tumours of the greater curvature and LESS—tumours of the lesser curve), and circular localisation (M—tumours in the distal two-thirds of the gastric corpus and L—tumours in antrum or pylorus).

The primary endpoint of this study was to evaluate the factors determining the prevalence of LNMs. The evaluation of factors was performed to identify the differences between T1a and T1b cancers and between node-negative and LNM patients.

Data are expressed as means ±standard deviations (±SD) or as percentages. Data were analysed using GraphPad Prism Software (GraphPad, San Diego, CA, USA). A comparison of means ±SD was performed with the two-tailed *t*-test. A univariate analysis was performed on all potential factors using the two-tailed chi-square test or Fisher’s exact test for categorical data and the ANOVA test for continuous data in groups larger than two. Multivariate logistic regression was performed by constructing models that took into consideration potential factors with a *p* value < 0.25 in the univariate analysis, according to the Hosmer–Lemeshow rule. A *p* value < 0.05 was considered statistically significant.

## 3. Results

This study involved 129 patients. Their demographic and clinicopathological characteristics are reported in Table 1.

The median age of the enrolled patients was 59.7 ± 10.8 years (range = 35–78 years).

The average size of the tumour was 2.9 ± 1.2 cm (range = 1.0–7.5 cm). Larger tumour sizes, depressed forms, ulceration, diffuse types, undifferentiated lesions, and GRE tumours, were seen in younger and male patients.

Greater tumour sizes were observed in T1b EGC and in LNM patients, rather than in T1a cancer and in node-negative patients (Table 2).

All patients received subtotal or total gastrectomy with D2 LN dissection depending on tumour location. Gallstones were documented in 11 (8.5%) patients.

LN dissection of the splenic hilum involved splenectomy in 32 (24.8%) cases. In all patients, the mean total nodal yield was 40.4 ± 5.3 (range = 31–62).

LNM was detected in 76 (59.0%) patients. Among T1 patients, 35 (44.3%) had LNMs, while in T1b patients, the incidence of LNMs was 82.0% (*p* = 0.0001).

Table 3 shows the number of LNMs in relation to clinicopathologic variables in node-positive patients. The female sex is directly related to the number of LNMs.

Higher numbers of LNMs were documented in tumours > 3 cm, in depressed aspect, presence of ulceration, diffuse type, undifferentiated tumours, and L location. Higher numbers of LNMs were documented in T1b cancer and in patients undergoing subtotal gastrectomy.

### 3.1. Risk Factors in T1a and T1b Patients

Table 4 shows the clinicopathologic characteristics of these two groups of patients.

There was no significant difference between T1a and T1b patients in terms of age (59.8 ± 11.4 years vs. 59.3 ± 9.7 years, *p* = 0.4) and retrieved LNs (40.0 ± 5.3 vs. 40.8 ± 5.3, *p* = 0.3). Patients with T1b cancer had larger tumour sizes (3.5 ± 1.3 cm vs. 2.5 ± 0.9 cm, *p* = 0.0001) and more LNMs (2.4 ± 1.0 vs. 1.3 ± 0.5, *p* = 0.00001) than patients with T1a cancer. The diffuse histologic type was observed significantly more often in mucosal cancer (73.4%) than in submucosal cancer (32.0%). Undifferentiated forms (64.0% vs. 26.5%), depressed-type macroscopic appearance (58.0% vs. 16.4%), and ulceration (54.0% vs. 15.1%) were observed significantly more often in T1b cancer than in T1a cancer. All LNMs in T1a cancer were found to be N1.

There were no significant differences regarding age between patients with LNMs and those without LNMs, both in T1a cancer (57.6 ± 13.2 years vs. 61.6 ± 9.6 years, *p* = 0.1) and in the T1b group (58.4 ± 9.6 years vs. 63.7 ± 9.7 years, *p* = 0.1). Greater tumour sizes were observed in LNM patients, both in T1a cancer (3.3 ± 0.7 cm vs. 1.8 ± 0.5 cm, *p* = 0.00001) and in T1b cancer (3.8 ± 1.2 cm vs. 2.1 ± 0.8 cm, *p* = 0.0001).

There were no differences in the number of retrieved LNs between patients with and without LNMs in both T1a (N0 40.8 ± 6.2 vs. LNM 39.0 ± 3.7, *p* = 0.1) and T1b cancer (N0 39.9 ± 2.0 vs. LNM 41.1 ± 5.8, *p* = 0.2).

Univariate analysis indicated that the male sex, tumour size > 3.0 cm, depressed-type appearance, ulceration, undifferentiated type, and diffuse Lauren’s criteria were risk factors for LNM in T1a EGC. In T1b patients, univariate analysis showed that L location had an association with LNMs. In these patients, tumour size > 3.0 cm, depressed lesion, ulceration finding, and the diffuse type were more frequent in the LMN group (Table 5).

### 3.2. Risk Factors for LNMs

There were no significant differences between patients with and without LNMs in terms of circular and longitudinal tumour location and the number of retrieved LNs (40.7 ± 5.7 in node-negative patients vs. 40.1 ± 5.0 in LNM patients, *p* = 0.5).

Patients with LNMs were younger than those without LNMs (58.1 ± 11.3 vs. 61.9 ± 9.6, *p* = 0.02). Depressed form, ulceration, diffuse histological type, and undifferentiated lesions were more frequent in LNM patients than in the node-negative group (Table 6).

In node-negative patients (53 cases), there were no significant differences between the T1a and T1b groups regarding age (T1a 61.6 ± 9.6 years vs. T1b 63.7 ± 9.7 years, *p* = 0.2), size of the tumour (T1a 1.9 ± 0.5 cm vs. T1b 2.1 ± 0.8 cm, *p* = 0.2), and retrieved LNs (T1a 40.8 ± 6.2 vs. T1b 39.9 ± 2.0, *p* = 0.3). In these patients, there were no significant differences between the T1a and T1b groups for the considered variables (Table 7).

In the LNM group, there were no significant differences between T1a and T1b patients in terms of age (57.6 ± 13.2 years vs. 58.4 ± 9.6, *p* = 0.3), size of the tumour (3.3 ± 0.7 cm vs. 3.8 ± 1.5 cm, *p* = 0.05), and LNs retrieved (39.0 ± 3.6 vs. 41.0 ± 5.8, *p* = 0.07). In these patients, the female sex (*p* = 0.01), depressed form (*p* = 0.0006), ulceration (*p* = 0.002), diffuse type (*p* = 0.0004), and undifferentiated lesions (*p* = 0.001) were more frequent in the T1b than in the T1a group.

Multivariate analysis revealed that tumour size greater than 3.0 cm, submucosal invasion, poor tumour differentiation, ulceration, and the diffuse type were risk factors associated with LNMs. Also, lower third location was a risk factor for LNMs (Table 8).

## 4. Discussion

EGC has a generally excellent prognosis, with the 5-year survival rate exceeding 90% after curative resection [2,3,5,10,16,19,29,31,32,33,34,35,36]. This scenario can be changed by LNMs, because node-positive patients show a significant decline in survival expectancy [3,16]. LNM is a critical factor for determination of therapeutic modalities [3,9,15].

In the past 20 years, D2 lymphadenectomy was considered to be the most appropriate therapy for EGC not amenable to less invasive treatment to achieve curative resection, where the LNM rate ranges from 10% to 42% [3,10,23]. This means that multiple patients underwent unnecessary LN dissection. Consequently, the clinicopathological risk factors for LNM are the key to the surgery rationale in EGC.

The present study found a relatively high LNM incidence in patients with EGC, which was notably higher than that of the Japanese cohort. Despite the different rates of LNM, analyses of risk factors for LNM revealed a similar finding with those reported in the literature. It was found that there were no significant differences in age or gender in EGC with or without LNM. However, a more aggressive biological behaviour and a higher risk of LNM were more frequently showed in younger patients [37]. In fact, larger tumour sizes and higher numbers of LNMs were seen in younger patients in this study. It was also indicated that the female sex was an independent risk factor for LNM in EGC patients [24]. In female patients, the biological behaviour of gastric cancer might be more aggressive, and this may not be fully dependent on tumour size. We noted that tumour sizes were greater in male rather than in female patients (*p* = 0.02), while higher numbers of LNMs were found in female patients than in male patients (*p* = 0.04). A possible explanation for the fact that gastric cancer tends to be more invasive in females could be related to endogenous oestrogen levels, which might promote tumour growth.

It has been suggested that ulcerative findings, a depressed appearance, diffuse type, and undifferentiated lesions are risk factors for LNM in EGC [5,8,13,17,22]. Depressed types tend to have higher rates of LNM in T1b cancer. The level of differentiation was an independent factor for LNM in EGC, with the rate of LNM in the undifferentiated type higher (from 13.6% to 32.5%) than the undifferentiated type (from 6.5% to 17.0%). Furthermore, it has been found that the LNM rate is 0.9% in the intestinal type and 4.2% in the diffuse type [38]. Our results agree with the findings in the literature. It has been documented that presence of ulceration has no significant association with LNM [39]. On the other hand, a study suggested that ulceration was an independent factor for LNM in EGC. Ulcerations were present in all LMN cases in T1a cancer [40]. Moreover, we noted greater tumour sizes and higher numbers of LNMs in patients with ulceration. Along with an increase in the diameter of the tumour, the incidence of LNM in EGC rises, both in T1a and in T1b cancer. A larger tumour diameter (≥2 cm) independently predicted LNM in poorly differentiated-type EGC. Our finding does not differ from large series published by Eastern authors. We observed greater tumour sizes in the T1b group and in LNM patients. Tumour sizes were larger in N2 patients (4.7 ± 1.3 cm) than in N1 (3.3 ± 0.8 cm) and N0 patients (1.9 ± 0.5 cm). Moreover, we reported that the number of LNMs was higher in tumours > 3 cm (2.1 ± 1.0) than in smaller tumours (1.5 ± 0.5, *p* = 0.006).

Further risk factors include L or LESS location. In these areas, the submucosa is thinner, and the lymphatic vessels more widespread in the lamina propria of the mucosa [41]. It has been demonstrated that tumour location is a significant but not independent factor for LNM in undifferentiated EGC. Nam et al. studied 2524 patients with T1a cancer and found that the ratio of LNM in tumours located in the upper, middle, and lower third parts in patients were 1.4%, 2.3%, and 2.3%, respectively. However, there was not a significant difference [38]. We observed a greater number of LNMs in L tumours (2.1 ± 0.9) than in M tumours (1.4 ± 0.8, *p* = 0.001), with no differences between GRE (1.9 ± 1.0) and LESS locations (1.9 ± 0.9, *p* = 0.4). Our results are confirmed by other studies in the literature in which a marked increase in LN involvement in distal EGC has been observed [42].

In our patients, LMNs were detected only in perigastric stations. However, our study highlighted that tumours with N2 LNMs were submucosal with a diameter greater than 3.0 cm. Thus, we suggest that submucosal cancer with a diameter greater than 3.0 cm is a risk factor for LNM. It has been observed that LNM starts with deep mucosal infiltration and as the invasion depth increases, the rate of LNM rises [3,8,13,14,22,31,33]. Our results agree that depth of invasion is one of the most important risk factors for LNMs. In the present study, T1b cancer was associated with a higher number of LNMs than in mucosal invasion, despite the fact that no LNM from station 7 to 12 was observed in our patients. D1+ lymphadenectomy has been recommended in Eastern countries for EGC where alternative treatment is considered unlikely to be effective [5,25,34].

However, the optimal extent of extragastric LN stations that should be included in D1+ lymphadenectomy has not been established. For this reason, D2 lymphadenectomy is considered the standard treatment for EGC. The guidelines recommend removing a minimum of 16 LNs. The removal of at least 16 LNs would allow the N stage to be precisely defined. A lymphadenectomy of at least 16 LNs would not lead to an increase in postoperative mortality [6,13,25,30,34,42]. The number of retrieved LNs serves as prognostic factor for gastric cancer, despite the fact that the optimal number of retrieved LNs remains controversial. Usually, the number of LNs is related to the extent of surgical intervention, which is also related to the depth of tumour invasion. As the depth increases, the number of LNs also increases. In our study, all cases of EGC had a radical gastrectomy and the difference between the number of retrieved LNs in T1a and in T1b cancer was not significantly different (*p* = 0.3).

This study has some limitations. First, the sample size was small. Second, this study’s retrospective nature can lead to potential bias. Third, we excluded patients with adenocarcinoma of the upper third of the stomach (Siewert III cancer) from the evaluation. Although it is commonly accepted that these tumours should be treated in the same way as mid-distal gastric cancer, patients with tumours of the upper third of the stomach were not included in the present study because the lymphatic flow in these cases is directed towards LN groups which are not included in D2 lymphadenectomy. Moreover, the depth of submucosal invasion was not specified in the present study. We demonstrate that several factors are related to LNMs in EGC. The frequency of LNM increases with the depth of invasion and a formal lymphadenectomy is necessary for the treatment of EGC. However, considering the limited research regarding a more tailored approach in the treatment of distal EGC, this study provides promising results. This preliminary work will need to be followed by further research concerning the extent of lymphadenectomy, with prospective and multicentre studies.

## 5. Conclusions

Our study revealed a relatively high incidence of LNM in EGC compared with Japanese and Korean cohorts. Large tumour size, undifferentiated type, ulceration, diffuse histological form, lower third location, and submucosal invasion were recognised as risk factors for LMN in EGC. In these patients, a radical gastrectomy with lymphadenectomy should be performed.

## Figures and Tables

**Table 1:** Demographic and clinicopathological characteristics in all patients.

Variables *		N. of Patients	%
Age	≤65 years	81	62.7
	>65 years	48	37.3
Sex	Male	71	55.0
	Female	58	45.0
Tumour size	≤3.0 cm	75	58.1
	>3.0 cm	54	41.9
Macroscopic appearance	Depressed	42	32.5
	Elevated	87	67.5
Ulceration	Absent	90	69.7
	Present	39	30.3
Lauren criteria	Diffuse	55	42.6
	Intestinal	74	57.4
Differentiation	Differentiated	76	58.9
	Undifferentiated	53	41.1
Circular location	M	40	31.0
	L	89	69.0
Longitudinal location	GRE	54	41.9
	LESS	75	58.1
Depth	T1a	79	61.2
	T1b	50	38.8
Surgery	Subtotal gastrectomy	94	72.8
	Total gastrectomy	35	27.2
N status	N-	53	41.0
	LNM	76	59.0
N stage	N0	53	41.0
	N1	62	48.0
	N2	14	11.0

* GRE: greater curvature; L: lower third; LESS: lesser curvature; LNM: lymph node metastasis; M: middle third.

**Table 2:** Tumour size in relation to the clinical, anatomical, and pathological parameters considered in all patients.

Variables *		Size (cm)	*p* Value
Age	≤65 years	3.1 ± 1.2	0.02
	>65 years	2.6 ± 1.1	
Sex	Male	3.1 ± 1.2	0.02
	Female	2.7 ± 1.2	
Macroscopic appearance	Depressed	3.6 ± 1.3	0.00001
	Elevated	2.6 ± 0.9	
Ulceration	Absent	2.6 ± 1.0	0.00001
	Present	3.6 ± 1.4	
Lauren criteria	Diffuse	3.4 ± 1.1	0.00001
	Intestinal	2.5 ± 1.1	
Differentiation	Differentiated	2.5 ± 0.9	0.00001
	Undifferentiated	3.6 ± 1.3	
Circular location	M	2.7 ± 1.0	0.1
	L	3.0 ± 1.3	
Longitudinal location	GRE	3.1 ± 1.3	0.04
	LESS	2.8 ± 1.1	
Depth	T1a	2.5 ± 0.9	0.00001
	T1b	3.5 ± 1.3	
Surgery	Subtotal gastrectomy	2.9 ± 1.2	0.3
	Total gastrectomy	2.8 ± 1.0	
N status	N-	1.9 ± 0.5	0.00001
	LNM	3.6 ± 1.0	
N stage	N0	1.9 ± 0.5	0.00001
	N1	3.3 ± 0.8	
	N2	4.7 ± 1.3	

* GRE: greater curvature; L: lower third; LESS: lesser curvature; LNM: lymph node metastasis; M: middle third.

**Table 3:** Number of LNMs in relation to the clinicopathological parameters considered in node-positive patients.

Variables *		N. of LNMs	*p* Value
Age	≤65 years	1.8 ± 0.9	0.09
	>65 years	2.1 ± 1.0	
Sex	Male	1.7 ± 0.8	0.04
	Female	2.1 ± 1.0	
Tumour size	≤3.0 cm	1.5 ± 0.5	0.006
	>3.0 cm	2.1 ± 1.0	
Macroscopic appearance	Depressed	2.6 ± 0.9	0.00001
	Elevated	1.3 ± 0.4	
Ulceration	Absent	1.3 ± 0.5	0.00001
	Present	2.6 ± 0.9	
Lauren criteria	Diffuse	2.1 ± 0.9	0.002
	Intestinal	1.5 ± 0.9	
Differentiation	Differentiated	1.3 ± 0.5	0.00001
	Undifferentiated	2.3 ± 0.9	
Circular location	M	1.4 ± 0.8	0.001
	L	2.1 ± 0.9	
Longitudinal location	GRE	1.9 ±1.0	0.4
	LESS	1.9 ± 0.9	
Depth	T1a	1.3 ± 0.5	0.00001
	T1b	2.4 ± 1.0	
Surgery	Subtotal gastrectomy	2.1 ± 0.9	0.008
	Total gastrectomy	1.5 ± 0.8	

* GRE: greater curvature; L: lower third; LESS: lesser curvature; LNM: lymph node metastasis; M: middle third.

**Table 4:** Demographic and clinicopathological characteristics of T1a gastric cancer compared with T1b gastric cancer.

Variables *		T1a	T1b	*p* Value
Age	≤65 years	45	36	0.09
	>65 years	34	14	
Sex	Male	45	26	0.5
	Female	34	24	
Tumour size	≤3.0 cm	57	18	0.0001
	>3.0 cm	22	32	
Macroscopic appearance	Depressed	13	29	0.0001
	Elevated	66	21	
Ulceration	Absent	67	23	0.0001
	Present	12	27	
Lauren criteria	Diffuse	58	16	0.0001
	Intestinal	21	34	
Differentiation	Differentiated	58	18	0.0001
	Undifferentiated	21	32	
Circular location	M	27	13	0.4
	L	52	37	
Longitudinal location	GRE	32	22	0.7
	LESS	47	28	
N status	N-	44	9	0.0001
	LNM	35	41	

* GRE: greater curvature; L: lower third; LESS: lesser curvature; LNM: lymph node metastasis; M: middle third.

**Table 5:** Risk factors for LNMs in T1a and T1b gastric cancer.

Variables *		T1a			T1b		
		LN-	LNM	*p* Value	LN-	LNM	*p* Value
Age	≤65 years	23	22	0.3	4	32	0.09
	>65 years	21	13		5	9	
Sex	Male	19	26	0.006	7	19	0.1
	Female	25	9		2	22	
Tumour size	≤3.0 cm	42	15	0.0001	7	11	0.006
	>3.0 cm	2	20		2	30	
Macroscopic appearance	Depressed	4	9	0.04	2	27	0.02
	Elevated	40	26		7	14	
Ulceration	Absent	41	26	0.02	7	16	0.03
	Present	3	9		2	25	
Lauren criteria	Diffuse	7	14	0.02	1	33	0.0002
	Intestinal	37	21		8	8	
Differentiation	Differentiated	37	21	0.02	9	9	0.0001
	Undifferentiated	7	14		0	32	
Circular location	M	15	12	1.0	6	7	0.005
	L	29	23		3	34	
Longitudinal location	GRE	18	14	1.0	2	20	0.2
	LESS	26	21		7	21	

* GRE: greater curvature; L: lower third; LESS: lesser curvature; LN: lymph node; LNM: lymph node metastasis; M: middle third.

**Table 6:** Univariate analysis of risk factors for LNMs in EGC.

Variables *		LN-	LNM	*p* Value
Age	≤65 years	27	54	0.02
	>65 years	26	22	
Sex	Male	26	45	0.2
	Female	27	31	
Tumour size	≤3.0 cm	49	26	0.0001
	>3.0 cm	4	50	
Macroscopic appearance	Depressed	6	36	0.0001
	Elevated	47	40	
Ulceration	Absent	48	42	0.0001
	Present	5	34	
Lauren criteria	Diffuse	8	47	0.0001
	Intestinal	45	29	
Differentiation	Differentiated	46	30	0.0001
	Undifferentiated	7	46	
Circular location	M	21	19	0.08
	L	32	57	
Longitudinal location	GRE	20	34	0.4
	LESS	33	42	
Depth	T1a	44	35	0.0001
	T1b	9	41	

* GRE: greater curvature; L: lower third; LESS: lesser curvature; LN: lymph node; LNM: lymph node metastasis; M: middle third.

**Table 7:** Univariate analysis of risk factors for LNMs in EGC in relation to depth of invasion.

Variables *		LN-			LNM		
		T1a	T1b	*p* Value	T1a	T1b	*p* Value
Age	≤65 years	23	4	0.7	22	32	0.2
	>65 years	21	5		13	9	
Sex	Male	19	7	0.07	26	19	0.01
	Female	25	2		9	22	
Tumour size	≤3.0 cm	42	7	0.1	15	11	0.1
	>3.0 cm	2	2		20	30	
Macroscopic appearance	Depressed	4	2	0.2	9	27	0.0006
	Elevated	40	7		26	14	
Ulceration	Absent	41	7	0.1	26	16	0.002
	Present	3	2		9	25	
Lauren criteria	Diffuse	7	1	1.0	14	33	0.0004
	Intestinal	37	8		21	8	
Differentiation	Differentiated	37	9	0.3	21	9	0.001
	Undifferentiated	7	0		14	32	
Circular location	M	15	6	0.1	12	7	0.1
	L	29	3		23	34	
Longitudinal location	GRE	18	2	0.4	14	20	0.4
	LESS	26	7		21	21	

* GRE: greater curvature; L: lower third; LESS: lesser curvature; LN: lymph node; LNM: lymph node metastasis; M: middle third.

**Table 8:** Multivariate analysis of risk factors for LNMs in EGC.

Variables *		OR	Wald	95% CI	*p* Value
Tumour size	≤3.0 cm	14.005	19.455	1.467 3.812	0.001
	>3.0 cm				
Ulceration	Absent	1.047	0.289	−1.064 1.868	0.003
	Present				
Lauren criteria	Diffuse	0.151	4.684	−3.604 −0.178	0.03
	Intestinal				
Differentiation	Differentiated	13.439	6.564	0.611 4.586	0.01
	Undifferentiated				
Circular location	M	0.062	4.995	−5.207 −0.341	0.02
	L				
Depth	T1a	15.034	6.957	0.696 4.724	0.008
	T1b				

* L: lower third; M: middle third; OR: odds ratio; CI: confidence interval.

## Data Availability

The authors confirm that the data supporting the findings of this study are available within the article.
